# Salinity‐induced transcriptome profiles in marine and freshwater threespine stickleback after an abrupt 6‐hour exposure

**DOI:** 10.1002/ece3.9395

**Published:** 2022-10-17

**Authors:** Annette Taugbøl, Monica Hongrø Solbakken, Kjetill S. Jakobsen, Leif Asbjørn Vøllestad

**Affiliations:** ^1^ Department of Biosciences, Centre for Ecological and Evolutionary Synthesis (CEES) University of Oslo Blindern Norway; ^2^ Norwegian Institute for Nature Research (NINA) Lillehammer Norway

**Keywords:** functional genomics, gene expression, osmoregulation, phenotypic plasticity, salinity tolerance

## Abstract

Saltwater and freshwater environments have opposing physiological challenges, yet, there are fish species that are able to enter both habitats during short time spans, and as individuals they must therefore adjust quickly to osmoregulatory contrasts. In this study, we conducted an experiment to test for plastic responses to abrupt salinity changes in two populations of threespine stickleback, *Gasterosteus aculeatus*, representing two ecotypes (freshwater and ancestral saltwater). We exposed both ecotypes to abrupt native (control treatment) and non‐native salinities (0‰ and 30‰) and sampled gill tissue for transcriptomic analyses after 6 h of exposure. To investigate genomic responses to salinity, we analyzed four different comparisons; one for each ecotype (in their control and exposure salinity; (1) and (2), one between ecotypes in their control salinity (3), and the fourth comparison included all transcripts identified in (3) that did not show any expressional changes within ecotype in either the control or the exposed salinity (4)). Abrupt salinity transfer affected the expression of 10 and 1530 transcripts for the saltwater and freshwater ecotype, respectively, and 1314 were differentially expressed between the controls, including 502 that were not affected by salinity within ecotype (fixed expression). In total, these results indicate that factors other than genomic expressional plasticity are important for osmoregulation in stickleback, due to the need for opposite physiological pathways to survive the abrupt change in salinity.

## Graphical abstract

This study has exposed wild caught sticklebacks from saltwater and freshwater and exposed them to their opposite salinity for 6 hours and analyzed their gill's gene regulation.

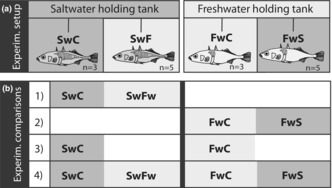

## 1 INTRODUCTION

How organisms are able to adapt to changing environmental circumstances is still a central problem in biology (Chevin et al., 2010; Delgado & Ruzzante, 2020), as many fine‐tuned mechanisms in one environment can be selected against in slightly different ecological settings. If possible, many organisms are able to use behavioral movements to avoid local stressful conditions (Sih et al., 2011; Wong & Candolin, 2015); fish can for instance avoid warm surface water by moving to deeper and colder areas, or evade fresher top layers in a fjord during snow melting in the spring. If movement is restricted, adaptive physiological responses may revert the organisms back to steady states within the new environment, in response to the new environmental cues, a phenomenon referred to as phenotypic plasticity (Pigliucci, 2001; Sangiao‐Alvarellos et al., 2005). Although sounding like an optimal evolutionary force on the short scale, there is an ongoing debate whether phenotypic plasticity is hindering or facilitating genetic adaptation in the long term (Ghalambor et al., 2015); because plasticity may change the phenotypes available for selection after exposure to a new environment, and consequently influence further genetic adaptations. Furthermore, plastic responses can be either adaptive or nonadaptive (Svensson et al., 2020) with respect to the local phenotypic optimum, and is generally assumed to have an influence on evolutionary trajectories through the altered distributions of phenotypes (e.g., expressional profiles) upon which selection can act. Having a wide possibility for plastic responses is often viewed as beneficial for adaption to new environments, as this gives a higher chance of expressing a “new phenotypic optima” directly, which then can be genetically assimilated in the new environment (Levis & Pfennig, 2016; Waddington, 1961), that is, the induced expression pattern in the novel environment will become “fixated”. Fixating environmentally induced plasticity through genetic assimilation should hence reduce genetic and plastic diversity in the derived population, where the rate of stabilizing selection depend on the number of loci that contribute to the additive genetic variance of the character(s) (Lande, 1976). Expression patterns of transcriptomes have been recognized as being plastic (Evans, 2015), implying that genetically similar individuals can have different transcriptome profiles (phenotypes) as a response to environmental cues (Mäkinen et al., 2016; Papakostas et al., 2014). Indeed, changes in gene expression can evolve very rapidly in many species, including fish (Roberge et al., 2008) and could, therefore, play an important role in the early steps of population divergence (Wolf et al., 2010).

For aquatic organisms such as fish, the difference between saltwater and freshwater represents considerably different selective forces. Most fish cells maintain a constant ion concentration, and few species are able to cross the salinity gradient between fresh and salt water (Delgado & Ruzzante, 2020). Most fish are therefore stenohaline, where the osmoregulatory machinery only operates within relatively narrow salinity boundaries (Hoar & Randall, 1984). Only about 3–5% of all fish species are euryhaline, meaning that they possess physiological mechanisms that allow them to adjust to a wide range of salinities (McCormick et al., 2012). Shortly, in saltwater, a fish will have a lower concentration of inorganic ions and hence a lower osmotic pressure compared to the environment, and the fish will passively gain ions and loose water (Evans et al., 2005; Rankin & Jensen, 1993). The situation for a freshwater fish is reversed, as the fish now has a higher concentration of ions when compared to the surroundings, and the fish passively gain water and loose inorganic ions. Consequently, to maintain homeostasis, saltwater fish drink saltwater, where excessive salts are actively secreted at the gills and water is absorbed in the intestine (Evans et al., 2005; Hoar & Randall, 1984), and freshwater fish actively absorb ions at their gills, minimize ion loss at their body surfaces, and actively reabsorb ions in their kidney to minimize urinary ion loss (Evans et al., 2005). Altogether, the cost of osmoregulation is highly variable, depending on salinity, oxygen, and temperature (Ern et al., 2014), where the total cost ranges from a few percent up to 30–50% of the total energy budget (Boeuf & Payan, 2001; Ern et al., 2014). In total, about 7% of the total energy budget can be spent in the gill tissue alone (Mommsen, 1984).

The threespine stickleback *Gasterosteus aculeatus* (hereafter stickleback) is a small fish known to have a wide salinity tolerance (Bell & Foster, 1994) at a seemingly low osmoregulatory cost (Grøtan et al., 2012). Originally of marine origin (Bell & Richkind, 1981), the stickleback has invaded and established populations in numerous freshwater habitats since the last glaciation in the northern hemisphere (Bell & Foster, 1994). Thus, stickleback populations are found at a wide osmotic range, spanning marine oceanic ecosystems, costal brackish water systems, freshwater rivers and lakes. Many populations have become landlocked after freshwater colonization, typically due to isostatic uplifting of the land following deglaciation, often restricting the gene flow between the founders and the derived populations. With reduced gene flow, and with freshwater habitats having a stable salinity compared to coastal waters, one would expect strong directional selection on traits that facilitate local adaptation to low salinity. Furthermore, one would also expect traits promoting a broad salinity tolerance (being euryhaline) to be selected against, due to the cost of sustaining characters that have not been required for many generations, and the low genetic variation typically found in derived populations (Schultz & McCormick, 2012). Genetic comparisons of marine and freshwater stickleback populations show signs of strong selection, and several outlier loci are identified by comparing whole genome sequences (Jones et al., 2012), SNPs (Guo et al., 2015; Hohenlohe et al., 2010; Jones, Chan, et al., 2012), and microsatellite genotypes (DeFaveri et al., 2011; Taugbøl, Junge, et al., 2014). Genetic studies further indicate that the frequency of freshwater‐linked alleles can increase rapidly in newly colonized freshwater habitats (Lescak et al., 2015). However, with respect to gene expression and potential gene regulatory adaptations in response to salinity, previous experiments have either tested candidate genes (McCairns & Bernatchez, 2010; Taugbøl, Arntsen, et al., 2014); compared populations directly without exposure to non‐native environments (Jones et al., 2012; Rastorguev et al., 2018); or tested for transcriptomic expression differences after a longer period of acclimatization in the non‐native environments (30 days to 3 months; Gibbons et al., 2017; Wang et al., 2014). However, less is known of the immediate expressional patterns following acute exposure to contrasting salinities. The objective of this study was to assess transcriptomic expression and compare regulatory changes in genes between marine and freshwater sticklebacks subjected to abrupt salinity transfers.

## 2 MATERIALS AND METHODS

### 2.1 Study sites, fish collection, and maintenance conditions

**FIGURE 1:**
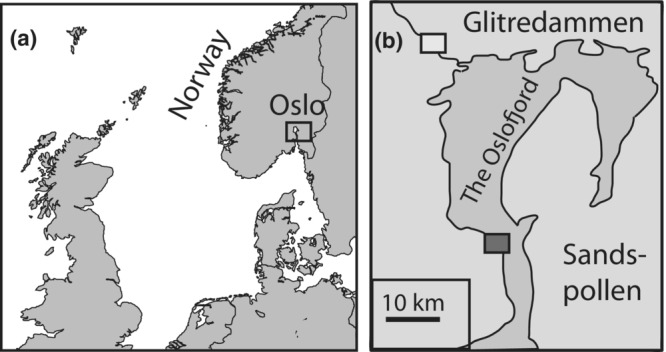
Study area. (a) map of Norway showing the position of the sampling sites just south of Oslo, (b) illustrates the two sampling locations: Glitredammen (freshwater) and Sandspollen (saltwater).

Fish used for this experiment were also part of a candidate gene study, and more methodological details can be found in Taugbøl, Arntsen, et al. (2014), Taugbøl, Junge, et al. (2014). Adult sticklebacks were captured at two locations near Oslo, Norway (Figure 1), during May and June 2010. The marine site, Sandspollen, has a salinity varying between 22‰ and 29‰, while the freshwater pond, Glitredammen, is stable at 0‰. Coastal stickleback populations in Norway are considered to be purely marine, potentially with some gene flow from nearby freshwater populations (Klepaker, 1996). Fish from Sandspollen breed locally (are not anadromous). The two locations are geographically isolated by approximately 35 km (shortest distance through water) (Figure 1b), where about 8.5 km is through the river Sandvikselva that contains several steep waterfalls and dams. This makes downstream movement of fish from Glitredammen toward the marine sampling site possible, but upstream movement from the fjord impossible. The stickleback in Glitredammen has probably been separated from marine ancestors for at least 7000 years, as the age of the lake has been estimated to be 7800 years before present using the program Sealevel32 (Møller, 2003). The lake Glitredammen is located at 82.2 m above sea level.

After capture, the fish were acclimated to holding conditions in their native salinity (30‰ or 0‰) for minimum 3 weeks prior to the experiment. To reduce potential male nesting behavior, the tanks were not equipped with any environmental enrichment, leaving the tanks free of sand and vegetation. The temperature in the tanks was maintained at room temperature (about 20°C), and the light regime was set at a 12:12 light: dark‐cycle. The fish were fed two times a day with frozen red bloodworms throughout the acclimation period. More details on fish maintenance can be found in Taugbøl, Arntsen, et al. (2014).

### 2.2 Experimental design

**FIGURE 2:**
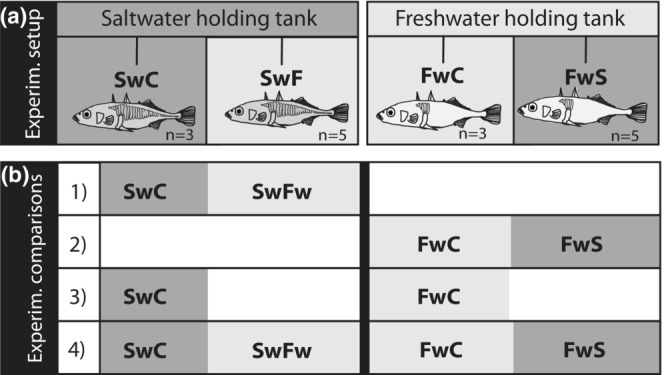
Experimental design. (a) Wild caught fish from saltwater and freshwater (see Figure 1) were taken into the laboratory and placed in holding tanks of their native salinity for a minimum of 3 weeks. After acclimation, two groups of eight fish from both populations/holding tanks were exposed to either saltwater (30‰; dark gray) or freshwater (0‰; light gray) for 6 h. The exposure tanks were divided in two by a perforated wall, so both populations could be exposed to the same water quality at the same time (SwC and FwSw shared tank, as did FwC and SwFw). (b) the four comparative analytical setups for which we compared the gene expression patterns: Comparison (1) saltwater control (SwC) compared to saltwater fish exposed to freshwater (SwFw); (2) freshwater control compared to freshwater fish exposed to saltwater (FwSw); (3) the two control groups; SwC compared to FwC; and (4) ecotype independent of salinity.

The experimental setup consisted of two 80‐L tanks (30‰ or 0‰), equipped with a gray plastic wall with punctures, dividing each experimental tank into two 40‐L compartments. At the start of the experiment, eight randomly selected fish that appeared healthy were collected from each acclimation tank and placed directly in separate, freshly prepared, experimental compartments. Each salinity/ecotype was tested in their native salinity; saltwater control (SwC) and freshwater control (FwC), and in their non‐native salinity; salt water ecotype exposed to freshwater (SwFw) and freshwater ecotype exposed to salt water (FwSw) (Figure 2). The fish were kept in the experimental tanks for 6 h before they were quickly netted out, immediately killed by a swift blow to the head, and directly processed for gill tissue sampling. Gills were used as they play an important role in the maintenance of blood ion and acid–base balance (Evans et al., 2005; Hoar & Randall, 1984). The experiment was approved by the Norwegian Animal experimentation and care committee (permit no ID 2705) and all efforts were made to minimize suffering. Large and sudden changes in salinity can influence survival and growth (Bachman & Rand, 2008); however, no fish died during the 6‐hour exposure, and results from the same experimental setup demonstrate very low mortality rates also when exposing fish for up to 3 weeks (Taugbøl, Arntsen, et al., 2014). Also, the same stickleback populations did not express differences in oxygen consumption rate after 14 days of exposure (Grøtan et al., 2012), indicating that long‐term salinity change has reverted the organisms back to steady states through adaptive physiological responses.

### 2.3 Tissue collection, RNA isolation, library preparation, and sequencing

After each fish was sacrificed, gill samples were immediately collected using sterilized tweezers and stored in RNA*later*® (Ambion® RNA; Life Technologies™). Of the 8 fish that were exposed in each experimental group, a total of three fish from each control (SwC and FwC) and a total of five fish from each exposure group (SwFw and FwSw) were processed for sequencing. For these 16 samples, messenger RNA (mRNA) was isolated from the gill tissue from each sample separately, using the mRNA direct kit with dynabeads (Invitrogen) as described by the manufacturer. The mRNA concentration and purity were quantified using an RNA 6000 Pico Kit on an Agilent 2100 Bioanalyzer (BioRad) according to the protocol, and all samples were diluted down to 0.125 μg/μl before cDNA synthesis. Libraries for RNAseq were prepared using the TrueSeg™ RNA low‐throughput protocol (Illumina), where all samples were fragmented for 4 min to obtain the required size distribution. All libraries were sequenced as 100 bp paired‐end at the Norwegian Sequencing Centre on the Illumina HiSeq 2000.

### 2.4 Aligning the gill transcriptome to the reference genome, filtering, and normalization of transcripts

The reads were mapped toward the *Gasterosteus aculeatus* genome with corresponding gene annotation (BROAD S1 Ensembl release 90, 2017) using bowtie2 v2.2.2 and tophat2 v2.0.14, and assembly of transcripts and expression performed using cufflinks v2.1.1. according to the Trapnell et al. protocol (Trapnell et al., 2012). All steps were done on the high‐performance computing cluster Abel at the University of Oslo (now replaced by the computing clusters hosted by Sigma2).

All analyses were run in R 4.0.1. (R Development Core Team, 2020) using the package edgeR (Robinson et al., 2010). First, the number of expressed reads for the individuals was compared as if an individual by chance was sequenced at greater depth, which might give this individual an unnaturally high gene expression count. The number of expressed reads before filtering varied from 3.832.109 to 25.515.142 (with an average of 8.756.949). The library sizes were adjusted by transforming the raw‐scale libraries to logged counts per million (CMP), before filtering out the genes with low expression values, as low read counts are a significant source of measurement error in differential expression analyses (Robinson & Smyth, 2007). Filtering out insignificant genes also increases detection power of significant discoveries (Bourgon et al., 2010). Therefore, a total of 597 genes (2.87%) that were unexpressed across all samples were excluded. We further excluded genes that was expressed at low levels across the individuals by the use of the function “FilterByExpr”; keeping genes with a CMP‐value of >0.68, and being expressed in at least three individuals, as that was the lower group size (Figure S1).

As small differences in expression of highly expressed genes between samples can give the appearance that many of the low‐expressed genes are differentially expressed between treatments, we normalized the read counts among libraries with the function “calcNormFactors”. This function uses the method of trimmed means of m values (TMM; Robinson & Oshlack, 2010) and normalizes the data by removing the extremely lowly and highly expressed genes, and also removes the genes that are very differentially expressed between samples, by keeping genes that was expressed at least six–seven times in the smallest sample, and being expressed in at least two of the libraries. The “calcNormFactors” then compares the total count for this subset of genes between the two samples and calculates a set of scaling factors for the library size that minimizes the log‐fold changes between samples for most genes, as the method assumes that the majority of genes are equally expressed between any two samples. The scaling factors in this study varied from 0.764 to 1.281 (Table S1). The calculated effective library size is then used as the original library size in all downstream analyses (Figure S2). Out of the 20.789 genes retrieved from the stickleback genome (Ensembl version 90), a total of 16.211 genes (77.9%) were kept for further analysis.

As the variance in RNA‐seq measurement of gene expression is typically overdispersed, a negative binominal distribution is used to model the variance. We calculated the common dispersion, using the same value for dispersion when modeling the variance for each gene, with the function “estimateCommonDisp,” and found the biological coefficient of variation to be 0.364, meaning that the true abundance for each gene can vary up or down by 36.4% between replicates. Each gene likely differs in dispersion, and the common dispersion model was extended to model the mean variance relationship between genes and the dispersion estimation per gene was calculated, shrinking the dispersion toward the trended dispersion due to low sample sizes, by the function “estimateGLMTagwiseDisp”. The results are visualized with a multidimensional scaling (MDS) plot, using the pairwise biological coefficients of variation as a distance measure to visualize the overall expressional relationships between individuals, and a principal component analysis (PCA) of the gene expression profiles using the *prcomp* function in R on log_2_‐transformed data.

### 2.5 Differential expression analysis between experimental groups

Differences in normalized transcript abundance levels were tested using a generalized linear model (glmQFit and glmQLFTest), with log_2_‐transformed transcript abundance as the response variable with a genome‐wide false discovery rate (FDR) of 0.05 with the function “*Toptags*”. The results of the gene expression differences are presented as centered and scaled heatmaps, plotted with gplots (Warnes et al., 2020). The groups that were compared was: comparison (1) saltwater control (SwC) versus saltwater ecotype exposed to freshwater (SwFw); comparison (2) freshwater control versus freshwater ecotype exposed to saltwater (FwSw); comparison (3) controls (FwC‐ SwC) and comparison (4) transcripts that are significantly different between controls, and similarly expressed within ecotype; all freshwater ecotypes versus all saltwater ecotypes (FwC_FwSw vs. SwC_SwFw) (Figure 2b). Comparison 3 identifies transcripts that are differentially expressed between the control groups, and to a lesser extent between comparison 1 and comparison 2, but where expression patterns *within* ecotype and salinity change do not differ *significantly*. Comparison 4 identifies genes that are similarly expressed within salinity of origin (the *ecotype*) independently of treatment. The transcripts in comparison 4 were extracted from the transcripts identified in comparison 3, by calculating the average expression for each transcript within each group (SwC, SwFw, FwC, FwSw) and extracting the genes that had <0.4 CMP differential expression between the respective control and exposed group. Overlapping genes in the four comparisons were identified and extracted with the package Venn diagrams (Gao, 2019).

### 2.6 Functional analysis of the differentially expressed genes: Gene ontology analysis

To identify potential biological functions that were overrepresented in the expressed genes, the differentially expressed up‐ and downregulated transcripts were extracted and tested for enriched Gene Ontology (GO) terms with the R package topGO 2.30.1 (Alexa et al., 2006) for biological processes (BP). The GO terms were extracted from the stickleback genome in Ensembl (n = 1829 GO‐terms that were linked to the 16.111 transcripts). Checking for significance, a classical Fisher test on GO‐terms with 10 or more annotated genes were used as a cut‐off. The top 10 results are presented as tables on sorted weighted values.

## 3 RESULTS

In this study, we performed transcriptome sequencing of individuals from two ecotypes of stickleback (saltwater and freshwater) being exposed to abrupt changes in salinity compared to their native environments. To investigate their overall response, we analyzed four different comparisons (outlined in Table 1): one for each ecotype, one between ecotypes in their control experimental salinity, and one aimed at finding transcripts that were equally expressed within ecotype regardless of salinity, through gene expression patterns.

**TABLE 1:** Number of transcripts for the different experimental comparisons, separated in up‐ and downregulated patterns (regulation), and the number of transcripts that were annotated to genes.

experimental Comparison	Regulation	Number of transcripts	Number annotated (%)
(1) SwC‐SwFw	Up in SwFw	3	3 (100)
	Down in SwFw	7	3 (42)
	Total	10	6 (60)
(2) FwC‐FwSw	Up in FwSw	691	569 (82.3)
	Down in FwSw	844	671 (79.5)
	Total	1535	1240 (80.7)
(3) SwC‐ FwC	Up in FwC	755	549 (72.7)
	Down in FwC	559	401 (71.7)
	Total	1314	950 (72.2)
(4) Ecotypes	Up in FW	329	265 (80.5)
	Down in FW	173	137 (79.2)
	Total	502	402 (80.0)

Abbreviations: SwC, saltwater control; SwFw, saltwater fish exposed to freshwater; FwC, fresh water controls; FwSw, freshwater exposed to saltwater.

### 3.1 Overall gene expression

**FIGURE 3:**
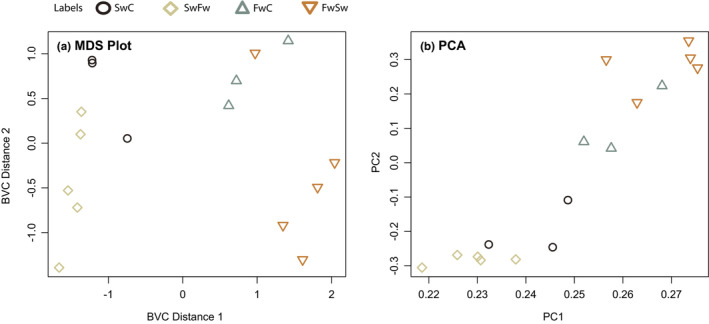
(a) MDS plot. Multidimensional scaling (MDS) plot generated with edgeR, where each point represents one sample and the distance between individual samples reflects the leading fold‐change (logFC) of the corresponding RNA samples. The leading logFC is the average (root mean square) of the 500 largest absolute logFCs for genes between those two samples (default plotting parameter) (two SC individuals are overlapping on the top), (b) principal component analysis (PCA) of gene expression profiles for all genes after filtering and normalization. FC, blue triangles pointing up; FS, orange triangles pointing down; SC, black circle's; SF, beige diamonds (see Figure 1 for abbreviations for groups).

When comparing all the 16.211 transcripts that were kept after filtration and normalization, the first axis of the multidimensional scaling (MDS) plot separated saltwater fish (SW) from freshwater fish (FW), whereas the second axis in part separated the exposure groups from their native salinity (Figure 3a). The Principal Component Analysis (PCA) on logged values of all expressed genes after filtration and normalization also separated the groups based on original salinity and treatment, where PC1 explained a total of 88.29% of the variation, and PC2 explained 1.88% (Figure 3b). Both the MDS and PCA separated the samples according to their original ecotype indicating that the overall gene expression pattern is highly linked to “original” environment.

**FIGURE 4:**
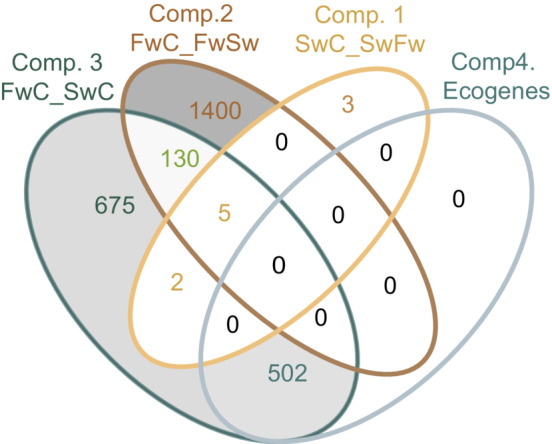
Venn diagram illustrating the total number of differentially expressed transcripts for the four different comparisons, see Figure 2 for abbreviations for groups.

**FIGURE 5:**
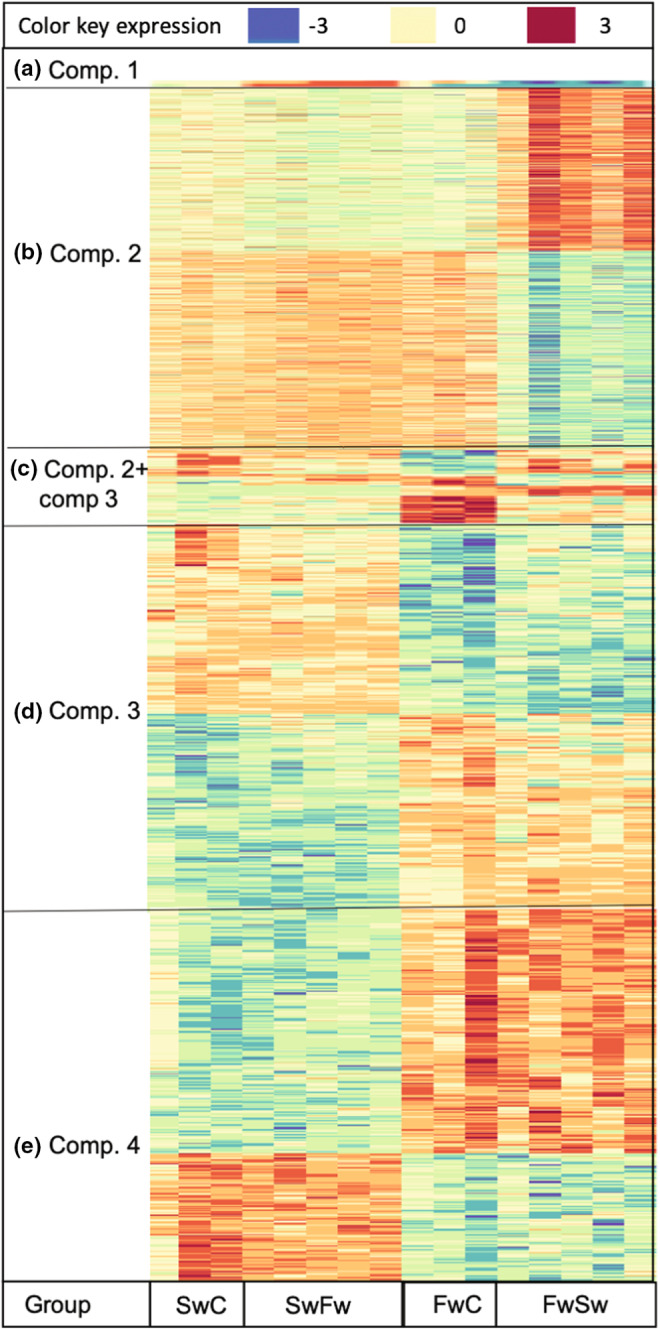
Heatmaps of the differentially expressed annotated genes from the different comparisons (comp), see Figure 2 for experimental comparisons and Figure 4 for the number of overlapping transcripts between the analyzed contrasts: (a) shows the results of all 10 transcripts that are significantly different in comparison 1, including the transcripts that overlap with comparison 2 and comparison 3; (b) shows the 1400 transcripts only significantly expressed in comparison 2; (c) shows the 130 transcripts that are significantly differentially expressed between both comparison 2 and comparison 3; (d) shows the 675 transcripts differentially regulated between comparison 3; and (e) shows the 502 transcripts that are different in comparison 4, the ecogenes. Blue color indicates less expression, red color indicates a higher expression.

In total, we identified 2717 unique transcripts that were significantly differentially expressed in all the analyzed contrasts (Table 1, Figures 4, 5, Table S1). Interestingly, comparing saltwater fish in salt‐ and freshwater only reported 10 differentially expressed transcripts (Figure 4, 5a). The contrast between freshwater fish in fresh‐ and saltwater gave the highest number of differentially expressed transcripts (~1500, Figures 4, 5b,c), followed by a comparison of the two controls (~1300, Figures 4, 5c,d) and differences in eco‐transcripts in salt and freshwater (~500; also being differentially expressed between the controls in comp. 3, Figures 4, 5e).

### 3.2 Genes differentially expressed in response to salinity (comparison 1 and 2)

**FIGURE 6:**
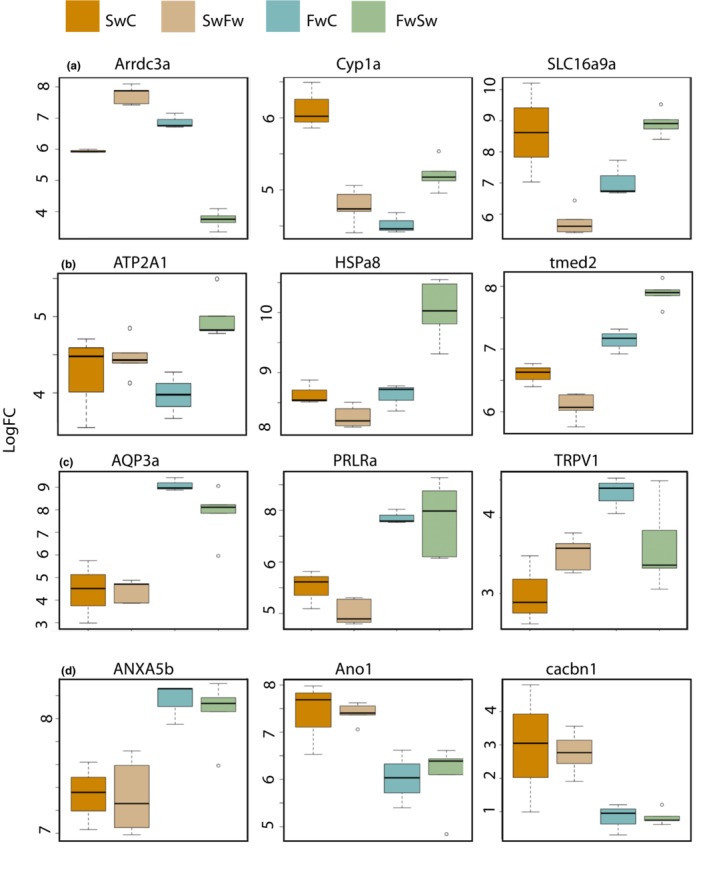
Examples of differentially expressed genes for the four different groups (SwC, SwFw, FwC, FwSW) plotted as boxplots, of (a) genes differentially regulated in comparison 1; (b) genes differentially regulated in comparison 2; (c) genes differentially regulated in comparison 3, and (d) genes differentially regulated in comparison 4. The boxplots show the 25–75% quantiles (boxes), median (black horizontal line), 95% limits (bars), and outliers (open circles).

When contrasting SwC and SwFw (comparison 1), a total of 10 transcripts were significantly differentially regulated, six which were annotated. Of the 10 transcripts, 3 transcripts were significantly different only for comparison 1 (Figure 4). Two of the transcripts were annotated; a gene predicted to be involved in the elongation factor process (*si:ch211‐13k12.2*) and the enzyme Galactosylceramide sulfotransferase (*GAL3ST1*). Five transcripts were shared with comparison 2: an arrestin domain (*arrdc3a*), a solute carrier (SLC16a9a) (Figure 6a), a cytochrome P450, family 1 (*CYP1a*) (Figure 6b), a docking protein (*dok4*), and a novel gene, *ENSGACG00000019379*, which share sequence homology with TBC1 family member 24 (BlastP GenBank) that has a potential function in intracellular trafficking. Two additional transcripts, which were also not annotated, were shared with comparison 3 (Figure 4).

Of the 1535 genes reported in comparison 2, between freshwater ecotypes, a total of 691 transcripts (569 annotated) were upregulated in FwSw when compared to FwC (Table 1). The upregulated gene list included several ion transporting ATPases; ATPase Na+/K+ transporting subunit beta 1a (*ATP1b1a*), ATPase sarcoplasmic/endoplasmic reticulum Ca2+ transporting 1 (*ATP2a1*, Figure 6b), and ATPase phospholipid transporting 11C (*ATP11c*); two ABC transporter genes were upregulated in FwSw (*ABCf2a* and *ABCb6a*) which belong to a family of proteins that utilize the energy of ATP binding and hydrolysis to transport various substrates across cellular membranes. A total of 20 different solute carriers were also upregulated in FwSw, and two genes involved in vesicular trafficking; transmembrane emp24 trafficking protein 2 (*tmed2*, Figure 6b) and 10 (*tmed10*). Genes linked to stress included five Heat Shock Protein (HSP) genes (*HSPa4b, HSPa5, HSPa8* (Figure 6b), *HSPa8b, HSPd1*) and seven ubiquitin specific peptidase (USP) genes. The *HSPa8* and *HSPa8b* encodes members of *HSP70*, and four DnaJ homologs which are co‐chaperones of the HSP70 family were also upregulated in FwSw (*dnaja1, dnajb1a, dnaja2a, dnajb9b*). Gene ontology enrichment of the upregulated genes involved processes such as peptide biosynthetic process, cellular response to xenobiotic stimulus, and glutathione metabolic process (Table 2).

**TABLE 2:** The top 10 gene ontology terms with significant number of annotated transcripts separated in upregulated and downregulated terms for biological processes

	GO.ID	Term	Annotated	Significant	Expected
Upp in FwSw	GO:0043043	Peptide biosynthetic process	283	15	12.54
	GO:0071466	Cellular response to xenobiotic stimulus	20	6	0.89
	GO:0006749	Glutathione metabolic process	28	7	1.24
	GO:0006364	rRNA processing	69	11	3.06
	GO:0044272	Sulfur compound biosynthetic process	47	8	2.08
	GO:0044262	Cellular carbohydrate metabolic process	57	9	2.52
	GO:0030835	Negative regulation of Actin filament de…	26	4	1.15
	GO:0019725	Cellular homeostasis	87	9	3.85
	GO:1901568	Fatty acid derivative metabolic process	23	5	1.02
	GO:0043065	Positive regulation of apoptotic process	25	5	1.11
Down in FwSw	GO:0006281	DNA repair	180	28	9.04
	GO:0006334	Nucleosome assembly	26	9	1.31
	GO:0060271	Cilium assembly	127	20	6.38
	GO:0034204	Lipid translocation	23	6	1.16
	GO:0090305	Nucleic acid phosphodiester bond hydroly…	105	11	5.27
	GO:0015914	Phospholipid transport	34	6	1.71
	GO:0006865	Amino acid transport	26	5	1.31
	GO:0033044	Regulation of chromosome organization	26	5	1.31
	GO:0016570	Histone modification	125	14	6.28
	GO:0006914	Autophagy	77	9	3.87

*Note*: The table shows results from comp. 2, were freshwater control (FwC) are compared to freshwater fish exposed to saltwater (FwSw) and in which direction the terms are regulated based on the FwSw fish. All terms significant with *p*‐values <.01.

A total of 671 annotated genes were downregulated in FwSw when compared to FwC (Table 1). Several known genes had reduced expression for freshwater fish when in saltwater; six genes related to ion‐transporting ATPases, including five related to phospholipid transporting (*ATP8a1, ATP8b2, ATP9a, ATP9b,* and *ATP10d*), and one linked to metallopeptidase and ATP synthase assembly factor homologs (*ATP23*). Furthermore, seven different solute carriers were downregulated, as were genes linked to the potassium voltage‐gated channel (*KCNd3*), the potassium inwardly rectifying channel (*KCNj2a*), a potassium channel tetramerisation domain (*KCTd12b*) and the chloride channel, voltage‐sensitive 6 (*CKCn6*). Two genes linked to calcium regulation; an EF‐hand calcium‐binding domain (*EFcab7*) and a transient receptor potential cation channel gene (*TRMP7*), and two genes linked to thyroid hormone signaling, thyroid hormone receptor interactor 10a (*trip10a*) and thyroid hormone receptor interactor 4 (*trip4*). Related to osmosensing, and involved in the activation and differentiation of immune cells, five interlukins all had similar expressions across groups, except for FwSw where they were downregulated (*ILf3a, ILf3b, IL113ra2, IL15, and IL19*). No HSP genes were downregulated, but one ATP‐binding cassette (*ABCh1*), three DnaJ homologs (co‐chaperones of the HSP70‐ family: *dnajc7, dnajc9,* and *dnajc11b*), together with three USP genes (*USP31, USP40, and USP45*) all had lower expression in FwSw. When testing all downregulated genes in this comparison, transcripts for GO enrichments processes such as DNA repair, nucleosome assembly, cilium assembly were included on top of the list (Table 2).

### 3.3 Genes differentially expressed between ecotypes (comp 3 and 4)

**FIGURE 7:**
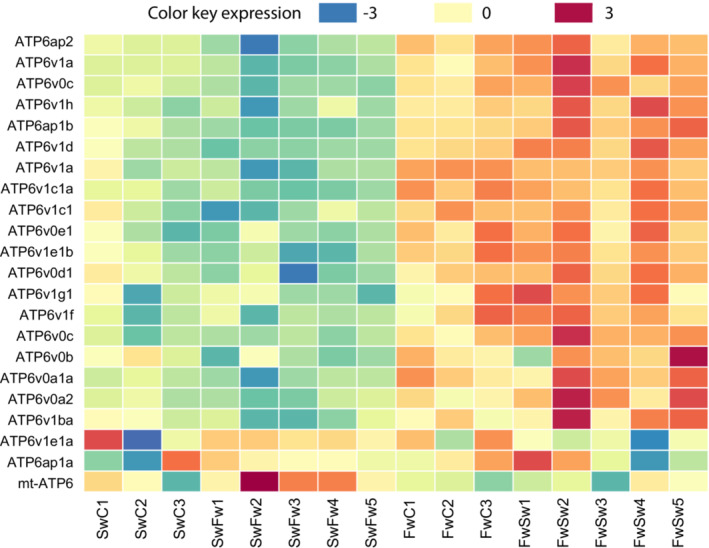
Heatmap of ATP6V0 and ATP6V1. For these two gene families, almost all transcripts showed signs of differential expression between the two ecotypes regardless of exposure treatment. The heatmap sums up individual fish on the X‐axis; Swc, saltwater control fish (*n* = 3); SwFw, saltwater fish exposed to freshwater (*n* = 5); FwC, freshwater control fish (*n* = 3) and FwSw, freshwater fish exposed to saltwater (*n* = 5).

In comparison 3 we only discuss differences between the two controls (Figure 2). Genes that were upregulated in SwC compared to FwC included the Sarcoplasmic/endoplasmic reticulum calcium ATPase 1 (*ATP2a1*), an enzyme that catalyzes the hydrolysis of ATP with the translocation of calcium from the cytosol to the sarcoplasmic reticulum lumen; several stress‐related cytochrome P450 (*CYP1a* [also overlapping with comparison 1 and 2], *CYP1c1, CYP1c2, CYP2y3, CYP7a1*), seven genes in the solute carrier family (including *SLC16a1b*), one myosin (*myh11a*), where the class XI myosins are associated with various organelles/vesicles (Tian et al., 2021). Genes upregulated in FwC compared to SwC included Aquaporin 3a (*AQP3a*), several ATPs (*ATP6V0C, ATP6V0C, ATP6v1d, AP6v1e1b*; Figure 7), arrestin domain containing 2 (*arrdc2*), 11 solute carriers, and prolactin Receptor type a (*PRLRa*) had a higher expression in freshwater, and a slight regulation within saltwater, as SwFw had a lower expression than SwC (Figure 6c **)**.

Of the 502 transcripts that had apparently similar expression within ecotype despite salinity change, 173 had a higher expression in the saltwater ecotype, and included one gene linked to ATP transport, ATPase phospholipid transporting (*ATP8a2*), two linked to calcium transport; calcium channel, voltage‐dependent (*cacnb1*, Figure 6d) and anoctamin 1, calcium‐activated chloride channel (*ano1*, Figure 6b), NADPH oxidase 1 (*nox1*), one solute carriers (*SLC6a1like*), interleukin 11 receptor (*IL11ra*) and genes linked to tyrosine kinase proteins, which in turn has been found to function as activators of several ion‐pumps; protein kinase 7 (*PTK7*), protein tyrosine phosphatase nonreceptor (*PTPN14*) and receptor type (PTP*rfa*), and erb‐b2 receptor tyrosine kinase 3b (*erbb3b*). A total of 329 transcripts had a higher expression in the freshwater ecotype, and included several ATPs (*ATP6V0e1, ATP6V1A, ATP6V1c1a, ATP6V1f*, Figure 7), where one is involved in T‐cell regulation (*tcirg1a*); four annexin genes, which are linked to salinity stress in plants (*ANXAa1, ANXA3b, ANXA5b* [Figure 6d] and *ANXA11b*), two linked to calcium: calcium homeostasis modulator family (*calhm5.2*) and mitochondrial calcium uptake (*micu2*) and one stress‐related UDP‐glucose (*ugp2b*). The freshwater ecotype also had a higher expression of claudins (*cldn1, cldn7b, cldnf)* and occludins (*oclnb*), proteins that are involved with tight junctions and reduction of ion efflux from the cells.

## 4 DISCUSSION

Here, we have investigated the transcriptomic response of an acute change in salinity for an allopatric freshwater and a saltwater ecotype of the threespine stickleback in Norway that have previously shown little energetic costs of salinity transfer (Grøtan et al., 2012). Within the 6‐hour exposure in this study, very few genes were significantly differentially expressed in saltwater stickleback transferred to fresh water (comparison 1), whereas about 1500 transcripts were differentially regulated in freshwater stickleback transferred to saltwater (comparison 2). Furthermore, over 1300 transcripts differed between the controls, including ~500 transcripts that did not express significant changes in regulation within salinity, but between ecotypes. These results indicate that the ability to adjust following a change in salinity is maintained by both ecotypes, but the gene expression cost of the transition seems much larger for the freshwater ecotype. As there were so few genes with significant plastic expression in contrast 1, we could not compare expression patterns toward higher or lower salinity for these ecotypes (salinity and ecotype interactions), and we could hence not find dominating gene groups equally important for abrupt salinity transfers in either direction. Many of the genes found to be differentially regulated within freshwater and between ecotypes in this study are known to be critical for ion regulation, as they facilitate transport through energy conversion, or are directly involved in building ion channels, ion pumps or suppress passive ion diffusion. Gills consists of different cell types, and some differentially expressed genes are involved in restructuring the gill tissue, through, for example, tightening the junctions between chloride cells in freshwater and likely increasing the density of the chloride cell type itself in saltwater (Perry, 1997). By also moving the control fish over to a new aquarium, the observed stress responses in this study should hence only relate to the salinity changes and the handling itself. Many genes in the HSP family, in addition to other stress‐related genes, were found to increase when freshwater fish were exposed to saltwater, but not for the saltwater fish when they were exposed to freshwater. Taken together, contrasting results between the two ecotypes strengthens the theory of many different evolutionary pathways to physiological freshwater adaptations in stickleback (DeFaveri et al., 2011; Gibbons et al., 2017) and other fish species (Velotta et al., 2017). Many genes linked to complex physiological regulatory mechanisms showed evidence of adapted expression profiles between the two ecotypes, supporting evolutionary adaptation via genetic assimilation and overall genomic reduction in phenotypic plasticity within the gill transcriptome, similar to findings in other fish species (Velotta et al., 2017).

### 4.1 Plastic gene expression profiles within ecotypes

A fish adapted to a particular salinity needs to have complex physiological regulatory mechanisms, at both the organismal and cellular level, in order to maintain water homeostasis. Changes in salinity will induce alterations in the nature and direction of ion transport, and genes linked to the maintenance of water homeostasis, cell signaling and structural permeability of cell membranes and stress responses, are likely targets of short‐term salinity responses. To start a physiological response, the fish must be able to recognize osmolarity change, where input from the osmolarity sensors also need to encode the magnitude, direction and ionic basis of the perceived change. Of the few genes with significant regulatory differences in both comparison 1 and 2, arrestin and SLC‐genes are linked to early osmosensory signal transduction. The solute carriers are membrane transport proteins mostly located in the cell membrane where they facilitate movement of small solutes across cell membranes in response to chemiosmotic gradients. A total of 27 SLC‐related transcripts were linked to salinity changes within the freshwater ecotype (contrast 2), including the Na^+^/H^+^‐exchanger (*SLC9a2*) with a higher expression in freshwater that also have been found in long‐term studies of freshwater acclimation in stickleback (although different isoforms; Gibbons et al., 2017). As expected, the Na^+^/K^+^/2CL^−^ cotransporter (*NKCC1*/ S*LC12a2*) had higher expression in saltwater, which also is consistent with findings in long‐term salinity exposures in stickleback (Gibbons et al., 2017) and other species of saltwater fish (Shaughnessy & McCormick, 2020). That *NKCC1* has a central role in salinity acclimation is further supported with no expression in gills of the salmonid grayling (*Thymallus thymallus*), which is a strict freshwater fish (Varadharajan et al., 2018). Three members of the monocarboxylate transporter family (MCT/*SLC16*) were upregulated in saltwater (Figure 6a), where *SLC16a9a* was one of the few genes differentially regulated in contrast 1. MCT's are involved in H^+^‐linked transport of monocarboxylic anions (Verri et al., 2012), that again are linked to the level of carnitine and energy metabolism by the transport of long fatty acid chains, like lactate, into mitochondria for energy production and between cell types. Fish gills are highly oxidative tissues, and oxygen requirements increase with increasing salinities (Vijayan et al., 1996), which again increase the natural concentrations of both plasma and gill‐cellular lactate (Mommsen, 1984; Sangiao‐Alvarellos et al., 2003, 2005). Lactate might hence be the primary candidate for rapid carbohydrate fuel in the gill tissue, especially for the saltwater fish in the early responses to reduced salinity.

Arrestin (*arrdc2, arrdc3a,* and *arrdc3b*) was downregulated in saltwater and *arrdc3a* was also included among the ten transcripts differentially regulated with salinity in saltwater fish (Figure 6a). Arrestins have been found to be involved in the modulation of diverse cellular processes through their adaptor functions, facilitating the localization and function of other proteins. Arrdc3a is linked to the GPCR regulation of the adrenergic signaling pathway, which again is linked to cellular Na^+^ regulation (Kumai et al., 2012), to increased insulin and glucose metabolism in mice livers (Batista et al., 2020), to growth in plants under salinity stress (Colaneri et al., 2014), and to stress and phosphorylation of the actin‐cytoskeleton in a soil amoeba, *Dictyostelium* (Habourdin et al., 2013). Arrestins have also been linked to positive activation of MAP kinases (Lefkowitz & Shenoy, 2005), a family of enzymes involved in osmosensory signal transduction (Fiol & Kültz, 2007), several of which are differentially regulated between comparisons 2 and 3 in this study. Arrestins have also previously been linked to other short time osmoregulatory experiments, being downregulated in turbot (*Scophthalmus maximus*) livers, when turbot acclimated to 30‰ salinity was exposed to 5‰ for 24 h (Cui et al., 2020), and four homologs of Arrestin was upregulated in the shrimp (*Halocaridina rubra*) (Havird et al., 2019) and crab (*Portunus trituberculatus*) (Lv et al., 2016) when they were transferred from 32‰ to 15‰ salinity, also for 24 h (similar results as in this study). That arrestins could have an important role in being “osmosensory genes” is also supported by DNA sequences for the arrestin gene *arrb2b*, as the sequences for stickleback and another euryhaline fish, the tiger pufferfish (*Takifugu rubripes*), were found to be more diverse from their alpha counterpart, *arrb2a*, than for several other fish species, which likely is a result of directional selection (Indrischek et al., 2017).

Several cytochrome P450 genes were upregulated in saltwater, including *CYP1a* that was one of the ten transcripts with differential regulation in contrast 1 (Figure 6a). *CYP1* is a superfamily of enzymes that catalyzes the oxidation of many reactions, and is widely used as an indicator of environmental pollution, also for stickleback (Knag & Taugbøl, 2013). The historic focus on *CYP1* as “only” a pollutant biomarker might have constricted the assessments of many related results to other potential pathways (Evans et al., 2005), as recent findings indicate a more direct link to general stress‐ and immune responses (Lenoir et al., 2021). The translation and expression pattern of *CYP1a* is being regulated by the aryl hydrocarbon receptor, *AHR*, which after heterodimerizing with *ARNT*, also is functional in immune cells of Atlantic salmon (*Salmo salar*) (Song et al., 2020), and when overexpressed, *CYP1a* has been found to actively suppress the expression of interferon type 1 (*IFNI*) (but *not IRF7*) in grass carp (*Ctenopharyngodon idella*), interferons that are secreted by infected cells (Chu et al., 2019). In previous salinity treatment experiments including fish, *CYP1a* has been found to be both upregulated and downregulated with salinity; Wang et al. (2014) found sticklebacks to have the highest expression in their original water quality (salt‐ and freshwater) when compared to freshwater fish in both 11‰ and 34‰ after 30 days of exposure (saltwater fish was only exposed to saltwater in this study), whereas *CYP1a* was found to be upregulated in tiger puffer after 30 days of exposure in the low salinity group (Jiang et al., 2020), and opposite, to increase with increasing salinities in coho salmon (*Oncorhynchus kisutch*) (Lavado et al., 2014) and rainbow trout (Leguen et al., 2010), as is similar to this study.

Maintaining cell volume is critical during salinity changes. Tight junction proteins such as claudins and occludins were upregulated in freshwater (comparison 4), similar to a long‐term salinity study on stickleback (Gibbons et al., 2017). Aquaporin 3a (*AQP3a*), a water channel protein linked to cell volume regulation and sensing, also had higher expression in freshwater, which is commonly found in euryhaline fish (Cutler et al., 2007; Velotta et al., 2017). In this study, AQP3a had a slight plastic chan*ge* within the freshwater ecotype, as FwS had lower expression than *FwC* (Figure 6c). Aquaporin expression has been found to be involved in the meditation of osmoreception in the tilapia prolactin secretion and gill chloride cell differentiation (Yan et al., 2013), and the DNA sequence for aquaporin in sticklebacks has previously been associated with positive selection between marine‐ and freshwater populations (DeFaveri et al., 2013 *;* Shimada et al., 2011), as has the gene expression patterns (Gibbons et al., 2017). Interestingly, in a purebred stickleback cross‐fostering experiment in 20‰ and 5‰, the expression pattern for *AQP3a* was equally expressed in the freshwater ecotype, and the saltwater ecotype had a higher expression with increased salinity (Hasan et al., 2017). This is the opposite pattern of what was found here. Wang et al. (2014) identified *AQP4*, another member of the aquaporin family, as a salt‐responsive gene in the kidneys of sticklebacks, although significant differences were only observed for freshwater fish in fresh‐ and saltwater (saltwater fish was only exposed to saltwater in Wang et al., 2014). In the present study, *AQP4* was filtered out due to low overall expression, but did have increased expression in the saltwater ecotype (data not shown).

Slow‐working hormones are involved in rearrangements during long‐term acclimation, by altering the abundance of ion transporters and cell proliferation, and differentiation of ionocytes and other osmoregulatory cells (Takei & McCormick, 2012). Prolactin is one of the slow‐working hormones, since long known to have a function in salinity acclimation (Pickford & Phillips, 1959). The concentration of Prolactin (PRLR) is typically increased following freshwater acclimation (Takei & McCormick, 2012) and more directly, prolactin has been linked to the chloride cell regulation; if injected with prolactin, the number of chloride cells in the gills of seawater acclimated tilapia decreases to the levels characterized by freshwater acclimated tilapia (Yan et al., 2013). Prolactin consists of two receptors; PRLRa and PRLRb, for which the expression patterns in gills have been found to be individually linked to salinity in tilapia (Fiol et al., 2009). Similar to this study, Fiol et al. (2009), we found the expression of PRLRa to be overall higher in freshwater (Figure 6c), with FwC expression being significantly different from both SwC and SwFw. In contrast, the PRLRb receptor had an increased expression in saltwater, but the difference was only significant in comparison2 (increased in FwSw). Specific activation of the two PRLR receptors was also found to activate different downstream signaling pathways, likely activating alternative routes leading to osmoprotection of gill cells during the period of active restructuring of gill epithelium in response to salinity stress (Fiol et al., 2009).

### 4.2 Salinity genes with contrasting ecotype expression profiles

Genetic assimilation occurs when a plastic ancestral trait becomes environmentally stable, resulting in a loss of plasticity (Lande, 1976). Many of the environmentally expressed genes were linked to known osmoregulatory and immune functions. Intracellular levels of calcium play an important role in responses to osmotic stress and functions in volume regulation of the cells (Erickson et al., 2001), and the ability to take up calcium at low Ca^2+^ concentration have likely been selected for in freshwater. Genes that were differentially expressed in salt‐ and freshwater linked to the uptake of Ca^2+^, and to a certain degree also for Na^+^ (Horng et al., 2007), included different ATP6Vs, subunits of a V‐type proton located at the basolateral membrane of mitochondria‐rich cells (Figure 7). Consisting of two main parts, the ATP6V1 comprise at least eight and the ATP6V0 include at least five different subunits (Sun‐Wada et al., 2004), where five V1 and two V0 subunits had a higher expression in freshwater fish in this study (contrast 3 and 4), clearly indicating that this gene has been important for both freshwater and saltwater acclimation, as was also found for killifish (*Fundulus heteroclitus*) (Whitehead et al., 2012). Other genes related to calcium were ano1 and cacbn1 (Figure 6d), both with an increased expression in saltwater fish.

Pathogen diversity in freshwater is often found to be higher than in salt water (Wang et al., 2012), and in teleosts the skin, gills, and gut are continuously exposed to the external aquatic environment and are, therefore, the main mucosal surfaces that represent potential entry ports for pathogens (Gomez et al., 2013). Many of the mechanisms for antigen sampling in the mucosal epithelium of teleost fish are mostly unknown, as they lack many of the mammalian molecules for transporting pathogens across the epithelia. Recent evidence suggests that two specific antigen‐sampling cell types exist in the gill, where one is expressing protein tyrosine phosphatase receptor type C (*PTPrc*) and IL‐1β (Kato et al., 2018). The PTPs catalyze the dephosphorylation of protein tyrosine kinases (*PTKs*) directly or through their downstream targets, and play key regulatory roles in multiple signal transduction pathways, where most are expressed in immune cells (Mustelin et al., 2005). In this study, increasing expression of PTPs were linked to high salinity, as two members of the PTP family were found to be differentially upregulated in the saltwater ecotype (*PTPN14* and *PTPrfa*), and three were upregulated in comparison 2 (FwSw; *PTPN2a, PTPN21, PTPrna*). In contrast, IL‐1β had overall higher expression in the freshwater ecotype. The second significant type of teleost antigen‐sampling cell types that were recently identified in gills was a microfold cell (M‐cell), expressing Anexin5 (*ANXA5*, Figure 6d) (Kato et al., 2018), a gene that has been linked to apoptosis by its ability to be recruited to the cell surface and co‐localize with phosphatidylserine; the “eat‐me” signal for macrophages (Lizarbe et al., 2013). In the present study, *ANXA5b* was significantly upregulated in the freshwater ecotype, which could indicate that the two different antigen sampling cell types might have been under directional selection in the opposite environments. *ANXA5* has also been linked to changes in calcium concentration, as they can bind around 12 Ca^2+^ ions and exhibit calcium channel activity in plasma membranes and in matrix vesicles (Lizarbe et al., 2013), so it is unclear if ANXA5 has a immunological or osmoregulatory function in freshwater fish (or both).

### 4.3 Concluding remarks

The saltwater fish in this study were collected from the Oslofjord, where they experience seasonal variation in salinity, due to periods of high freshwater influx from rivers after heavy rain and snow‐melting. It is, therefore, even more surprising that the saltwater fish exhibited such relatively low significant expressional plasticity when exposed to freshwater, although theoretical studies have shown that fluctuating environments can reduce plasticity (Leung et al., 2020). Furthermore, the genetic background for the saltwater fish is likely more diverse. Having a more diverse genetic makeup can likely also lead to a higher variation expression (higher standard deviations), which again will impact the false discovery rate and estimations of significance between experimental groups when filtering on expressional differences as in this study. However, recent studies suggest that a reduced level of genetic diversity can even increase the expressional diversity, and possibly buffer some of the loss of adaptive potential given with a higher genetic variation (Liu et al., 2019; Mazzarella et al., 2015; Morris et al., 2014). More comprehensive studies on physiological changes and osmoregulatory transcriptional responses are needed to understand how the stickleback, especially the saltwater stickleback, are able to tolerate short‐term salinity changes. The low genetic expressional differences for the saltwater fish in this study indicates that they invoke some alternative strategy than gene regulation to handle changing salinities, like reversing the orientation of the proteins in the cell membrane (Hartmann et al., 1989), changing the activity state and/or function of cells and cell types, or proteins after interacting with other proteins (Pertl et al., 2010; Szczesnaskorupa et al., 1988), and/or mitochondrial activity/morphology or numbers (Austin & Nowikovsky, 2021); as short‐term cellular adjustments are needed in order for the cell volumes to remain stable when moved abruptly from 30‰ to 0‰.

## AUTHOR CONTRIBUTIONS

**Annette Taugbøl:** Conceptualization (equal); formal analysis (lead); methodology (equal); project administration (lead); visualization (equal); writing – original draft (equal). **Monica H. Solbakken:** Data curation (equal); formal analysis (equal); writing – review and editing (equal). **Kjetill S. Jakobsen:** Conceptualization (equal); project administration (equal); writing – review and editing (equal). **Leif Asbjørn Vøllestad:** Conceptualization (equal); project administration (equal); supervision (lead); writing – review and editing (equal).

## Supporting information


Table S1

Table S2

## Data Availability

All data are available as supplementary material on the online version of this manuscript (Table S2), or by emailing the first author.
