# Supplementary material for: Salinity‐induced transcriptome profiles in marine and freshwater threespine stickleback after an abrupt 6‐hour exposure
Source: Ecol Evol. 2022 Oct 17;12(10):e9395. doi: 10.1002/ece3.9395 (PMC9596333; doi:10.1002/ece3.9395)
Supplement: Supplementary file 1 — Table S1 [file ECE3-12-e9395-s002.docx]

**Supplementary Taugbøl et al.**

**Supplementary Table 1.** Background information. Identification name (Id) for each fish, sallinity of origin (Org. salinity), experimental salinity (exp. Salinity), sex (males and females), raw library size (Library size Raw), efficient library size (Library size eff) and normalization factors. More information in materials and methods.

| **Id** | **Org. salinity** | **Exp. salinity** | **Sex** | **Library size_Raw** | **Library size_Eff** | **Norm. factors** |
| --- | --- | --- | --- | --- | --- | --- |
| FwC1 | Fresh | Fresh | F | 9956299 | 9947209 | 0.992 |
| FwC2 | Fresh | Fresh | M | 6230221 | 6225108 | 1.084 |
| FwC3 | Fresh | Fresh | F | 5959854 | 5956180 | 0.825 |
| FwSw1 | Fresh | Salt | F | 5279155 | 5276459 | 0.842 |
| FwSw2 | Fresh | Salt | F | 6928982 | 6923647 | 0.783 |
| FwSw3 | Fresh | Salt | M | 25515142 | 24647700 | 0.799 |
| FwSw4 | Fresh | Salt | M | 3832109 | 3828159 | 0.764 |
| FwSw5 | Fresh | Salt | M | 7529648 | 7522266 | 0.825 |
| SwC1 | Salt | Salt | F | 14760121 | 14748241 | 0.947 |
| SwC2 | Salt | Salt | M | 5547532 | 5540189 | 1.267 |
| SwC3 | Salt | Salt | M | 9940694 | 9928611 | 1.281 |
| SwFw1 | Salt | Fresh | F | 9430399 | 9410129 | 1.189 |
| SwFw2 | Salt | Fresh | F | 3953550 | 3934076 | 1.152 |
| SwFw3 | Salt | Fresh | F | 9948379 | 9936221 | 1.150 |
| SwFw4 | Salt | Fresh | F | 6494566 | 6488094 | 1.127 |
| SwFw5 | Salt | Fresh | F | 8804537 | 8787217 | 1.243 |

**Supplementary Figure 1**: Before and after filtering. a) the number of expressed reads before filtering varied from 3.832.109 to 25.515.142 (with an average of 8.756.949). The library sizes were adjusted by transforming the raw-scale libraries to logged counts per million (CMP), and transcripts were filtered out by the use of the function “FilterByExpr” in edgeR; keeping genes with a CMP-value of > 0.68, and being expressed in at least three individuals, as this is the lower group size (b).

**Supplementary Figure 2**: Before (a) and after (b) normalization. The read counts were normalized among libraries with the function “calcNormFactors”. This function uses the method of trimmed means of m values, and normalizes the data by removing the extremely lowly and highly expressed genes, and also removes the genes that are very differentially expressed between samples, by keeping genes that was expressed at least 6-7 times in the smallest sample, and being expressed in at least two of the libraries.
